# Acute Effects of Insulin on Cardiac Function in Patients with Diabetes Mellitus: Clinical Applicability and Feasibility

**DOI:** 10.1155/2020/8134548

**Published:** 2020-03-17

**Authors:** Deeb Daoud Naccache, Sergey Yalonetsky, Ronen Bar-Yoseph

**Affiliations:** ^1^Institute of Endocrinology, Diabetes and Metabolism, Rambam Health Care Campus, Haifa, Israel; ^2^Institute of Echocardiography and Cardiology, Rambam Health Care Campus, Haifa, Israel; ^3^Pediatric Pulmonary Institute, Ruth Children's Hospital, Rambam Health Care Campus, Haifa, Israel

## Abstract

**Background:**

Insulin promotes glucose consumption as the main cardiac energy source, while increasing myocardial efficiency. The short-term effects of insulin on cardiac function and its potential curative role in an acute diabetological cardiology setting remain unknown. Our study evaluated the role of acute insulin administration in the diabetic heart, its corresponding effective blood insulin level, and the time-course applicability of insulin treatment in a routine clinical setting.

**Methods:**

We evaluated a case series of six male (48.1 ± 4.9 y/o) patients with controlled diabetes (HbA1c of 6.6 ± 0.3%) and disease duration of 14.4 ± 6.7 yr. Each subject was evaluated for glucose homeostasis, as well as hemodynamic and *echocardiographic (systolic and diastolic) parameters* at *three points: baseline followed by* two successive insulin loads in euglycemic hyperinsulinemic clamp study. Results were analysed using Student's *t*-test.

**Results:**

The first insulin load led to a physiologic blood insulin level of 145 ± 36 *μ*U/ml, and both systolic (7 mmHg) blood pressure and diastolic (4 mmHg) blood pressure decreased significantly. Left ventricular fractional shortening (LVFS) increased significantly by 11.8%. Diastolic function parameters of mitral annulus movement of the A′ wave increased relative to baseline by 20.0% (27.8% under the second insulin load), A′ medial increased relative to baseline by 30%, and A′ lateral increased relative to baseline by 17%, displayed by tissue Doppler imaging.

**Conclusions:**

Insulin acutely affected the diabetic heart at a physiologic level within a 2 h time course. Insulin mainly increased left ventricular systolic function and, to a second degree, improved left ventricular diastolic functions and atrial systole in diabetic subjects. These results may facilitate the development of insulin-based acute treatment in diabetic patients with cardiac morbidity. This trial is registered with NCT02962921.

## 1. Introduction

Diabetic cardiomyopathy is diagnosed when ventricular dysfunction develops in patients with diabetes who are otherwise free of coronary atherosclerosis or hypertension [1, 2]. Diabetes is significantly associated with diastolic dysfunction in the absence of systolic dysfunction [3, 4]. Diabetic diastolic dysfunction is an important clinical entity [5] that has a particular clinical importance when it exists with risk factors for pulmonary congestion, e.g., events of tachyarrhythmia [6].

Hypertrophy and diastolic abnormalities are the most common and are observed earlier than systolic abnormalities in diabetic subjects. As such, diastolic abnormalities dominate clinical findings in type 2 and type 1 diabetes [7]. Atrial contraction during diastole is affected by serum glucose and insulin levels [8]. While a different pathophysiology underlies type 1 and type 2 diabetes mellitus, hyperglycemia per se bestows biochemical overlaps in both types of diabetes. A hypothesis proposed the contribution of decreased calcium flux into the cardiomyocyte combined with increased apoptosis, to diastolic dysfunction [7]. Moreover, increased oxidative metabolism had been speculated as an early event in diabetic cardiomyopathy in type 1 diabetes [9].

Insulin promotes glucose as the main cardiac energy source, and it reduces oxygen consumption, augments cardiomyocyte contraction, and increases cardiac efficiency [10]. Insulin had proved efficacious in various hyperglycaemic states, in absence and in presence of different comorbid states, over a spectrum of treatment duration:

(1) Upon myocardial function:
  - Improving left ventricular (LV) diastolic abnormalities [11]
  - Improving LV diastolic function [12] both in healthy and type 2 diabetes mellitus [13]
(2) Upon ischemic heart disease (IHD):
  - Having an anti-inflammatory and profibrinolytic effect in the setting of acute myocardial infarction (MI) both in diabetic and nondiabetic subjects [14]
  - Improving regeneration of ischemic myocard ensuing coronary bypass grafting in acute MI [15]

In the setting of IHD insulin effect was attributed to mainly reducing senescent myocyte precursor cells in the peri-infarction region, hence, increasing capability of ischemic myocard to regenerate. Insulin also improved left ventricle ejection fraction (LVEF) rates among nondiabetic patients in acute MI [12]; this protective effect was ascribed, ex-vivo, to the induction of cell-survival signalling via akt and p70s6 kinase [16]. Physiologically, insulin increased coronary blood flow and decreased coronary arteries resistance among healthy persons [17].

It is widely accepted that beneficial insulin effect on endothelium is to enhance eNOS expression. Whether it was tested among critically ill diabetic as well as nondiabetic patients [18] or in human aortic cells where insulin induced a dose-dependent induction of e-NOS, insulin may contribute to the overall vasodilatation [19].

The acute short-term effects of insulin administration on LV function (LVF), specifically those pertinent to atrial and ventricular functions during diastole, are unclear.

The aim of the current study was to investigate the following questions: (1) Does short-term insulin infusion affect diabetic heart function? (2) What is the corresponding effective insulin blood level? And (3) is the time course applicable in a clinical setting?

## 2. Materials and Methods

### 2.1. Patients

Six male, diabetic patients, 30−65 years old, with an established diagnosis of diabetes mellitus for at least 2 years, who were free of significant valvular heart disease, atrial arrhythmia, or cardiac pacing, comprised the study population. The characteristics of the patients are summarised in Table 1.

Each patient served as his own control. The study protocol was approved by the ethics committee at Rambam Medical Center, Haifa, Israel. Prior to the study, patients gave written informed consent after receiving a detailed explanation of the purpose of the study and the technique and its side effects and the study protocol.

Patients were studied within a clinical research facility at the Rambam Medical Center, Haifa, Israel. We conducted baseline evaluation at 8:00 am after 12 h of fasting, followed by two euglycemic hyperinsulinemic clamp (EHC) steps (2 h for each step). The vital signs monitored during the study protocol were manual heart rate, systolic blood pressure (SBP), diastolic blood pressure (DBP), and baseline 12-lead electrocardiogram (ECG) recording. Glucose consumption rates under changing insulin levels were measured by the EHC technique. Cardiac function parameters were measured in the last 10 min of each step by tissue Doppler echocardiography. Results are presented as the mean ± standard error of the mean (SEM).

### 2.2. Clamp Technique

Each patient underwent an EHC study, according to the protocol described previously [20, 21] and implemented at our institute [22]. Baseline measurements of blood insulin and glucose levels were collected. Thereafter, two insulin loads were initiated. The blood glucose level was kept near euglycemia (90 ± 5 mg/dl) throughout the 2 h period of each insulin load step. Intravenous (iv) insulin (Lispro Insulin, Eli Lilly, France) infusion rate was 1- milliunit per kg body weight per min (mU/kg·min) at step number one and 10 mU/kg·min at step number two of EHC study. Fifty percent dextrose in water (DW50) was infused at variable infusion rates and titrated to maintain euglycemia. Concomitant saline (0.45%) was used to dilute the DW50 to prevent hypertonicity-induced irritation of the cannulated vein. During the baseline period, saline (0.45%) infusion rates were 0.75 ml/min to keep the iv cannula open. Every patient was given a chance to urinate before Step 2. Glucose and insulin homeostatic parameters of the study group were compared to those of healthy controls that were studied earlier [23].

### 2.3. Cardiac Function Assessment

Transthoracic echocardiography was performed by a qualified echocardiographer using a General Electric Vivid 3 machine (Tirat HaCarmel, Israel). Parameters were assessed as the mean of three consecutive heart beats.

#### 2.3.1. Systolic Echocardiographic Parameters

LV fractional shortening (LVFS (*see note 1*)) was derived from end diastolic and end systolic LV dimensions; LV ejection fraction (LVEF (*see note 2*)) was derived from end diastolic and end systolic LV volumes [24, 25].

#### 2.3.2. Diastolic Echocardiographic Parameters

1. Mitral valve diastolic flow parameters (by pulse wave Doppler) were as follows: (a) *E* wave which represents early diastolic filling, (b) *A* wave which represents late diastolic filling concomitant with the atrial kick, (c) *E*/*A* ratio, and (d) *E* wave deceleration time (DT).
2. Mitral annulus (tissue Doppler-derived) velocities were as follows: (a) *E*′ mean, which represents early diastolic velocity (synchronous to the *E* wave), was derived from *E*′ medial (measured close to the interventricular septum) and *E*′ lateral (close to lateral wall). (b) The *E*/*E*′ ratio represents diastolic LV compliance. (c) *A*′ mean, which represents mitral annulus movement velocity (synchronous to mitral inflow *A* wave), was derived from *A*′ medial (measured close to the septum) and *A*′ lateral (measured close to lateral wall).

Results are presented as the mean ± SEM and analysed using Student's *t*-test. Statistical significance was considered when the *p* value was less than 0.05 [24, 25].

## 3. Results

### 3.1. Heart Rate, Blood Pressure, and Electrocardiogram

No significant change was observed in the heart rate throughout the study (Table 2). At Step 1 of the EHC, systolic blood pressure (SBP) decreased significantly from 138 ± 7.6 to 131 ± 9 mmHg (*p* < 0.05). Step 2 of the EHC caused a decrease to 126 ± 6 mmHg, but the change was not significant (NS).

At Step 1 of the EHC, diastolic blood pressure (DBP) decreased from 80 ± 5.6 to 76 ± 5 mmHg (*p*=0.05). No additional change was observed following Step 2 of the EHC (77 ± 6 mmHg) (Table 2). No changes were observed in the electrocardiogram throughout the study.

### 3.2. Insulin Infusion and Insulin Blood Levels

Baseline, Step 1, and Step 2 of the EHC generated blood insulin levels as summarised in Table 2. Step 1 of the EHC resulted in a physiologic 145 ± 36 micro International Units per millilitre (*μ*U/ml) blood level of insulin.

### 3.3. Glucose Consumption Rates

Step 1 of the EHC prompted a glucose infusion rate (GIR) of 79 ± 25 mg/min·m^2^, which was adequate to compensate for insulin-induced glucose disposal and maintain euglycemia. This is compared to a blood insulin level of 51 ± 5 *μ*U/ml with its respective GIR of 227 ± 24 mg/min·m^2^ in healthy subjects (Table 2).

Step 2 of the EHC generated a blood insulin level of 1863 ± 433 *μ*U/ml, which prompted a GIR of 165 ± 38 mg/min·m^2^. This is compared to a blood insulin level of 898 ± 131 *μ*U/ml, with its respective GIR of 478 ± 17 mg/min·m^2^ in the healthy group.

These insulin levels and their corresponding GIR indicated an expected degree of insulin resistance in the diabetic group compared with that of normal subjects [15], although the studied diabetic group had fair glycaemic control (HbA1c = 6.6 ± 0.3%).

### 3.4. Systolic Function

Baseline LVEF was within normal values for all study patients, at 67.6 ± 2.0% compared with 67% in healthy adults, according to the standard values in our echocardiography unit.

At Step 1 of the EHC, the LVEF was 73.9 ± 3.5% (9% increase; NS). Step 2 of the EHC drove an additional nonsignificant 1% increase (74.5 ± 3.8%, Table 3). The increase in LVEF from baseline to Step 2 of the EHC totalled 10% (NS).

The baseline LVFS was within normal values for all study patients, at 36.9 ± 1.6% compared with 18–42% in healthy adults, according to the standard values in our echocardiography unit (Table 3). At Step 1 of the EHC, the LVFS was 41.3 ± 2.1% (11.8% increase; *p* < 0.05). Step 2 of the EHC drove an additional nonsignificant 4.7% increase (43.0 ± 2.2%, Table 3). The increase in LVFS from baseline to Step 2 of the EHC totalled 16.5% (*p* < 0.05).

### 3.5. Diastolic Function

All diastolic function parameters (DT, *E* wave, *A* wave, *E*/*A* ratio, *E*′ mean, *E*′ lateral, *E*/*E*′ ratio), with insignificant changes under either insulin loading step, as well as those parameters with significant changes, are summarised in Table 3. *E*′ medial increased at Step 1 of the EHC by 7.5% (*p* < 0.03) and did not change at Step 2 of the EHC. *A*′ mean increased at Step 1 of the EHC by 20% (*p* < 0.01), and at Step 2 of the EHC it increased by 8% compared to Step 1 (*p* < 0.05), with a global change from baseline to Step 2 of 27.8% (*p* < 0.005). *A*′ medial increased at Step 1 of the EHC by 30% (*p* < 0.0005) and by 14% at Step 2 of the EHC (nonsignificant versus Step 1), with global change from baseline to Step 2 of 44% (*p*=0.005). *A*′ lateral increased at Step 1 of the EHC by 17% (*p*=0.003), and it did not change significantly at Step 2 of the EHC versus Step 1, with a global change from baseline to Step 2 of 17% (*p* < 0.05).

E did not change significantly throughout measurements during baseline, Step 1, and Step 2 of EHC (82.3 ± 8.6, 80.2 ± 8.7, 84.2 ± 10 cm/second, respectively). *E*/*E*′ also did not change significantly (7.8 ± 0.5, 7.5 ± 0.4, 7.6 ± 0.6, respectively).

### 3.6. Infusates

Table 4 summarises all intravenous infusates given during the 4 h clamp study. Insensible water loss was not calculated due to lack of room humidity measurements.

## 4. Discussion

In this pilot study, for the first time, we demonstrated an acute effect of physiologic insulin blood levels on blood pressure and left ventricular diastolic parameters, along with an already known positive effect on left ventricular systolic function. However, there are few differences from previous studies as different insulin loads applied (4.16 mIU/kg.min in acute MI [12], 1 mIU/kg.min in healthy men [17], and 41.6 mIU/min regardless of body weight in acute MI [14]) and a different time course of the insulin treatment, as short as 48 hrs [12, 14], three days [15], and one month period [11] vs the shortest two-hour time reported in our study.

Previous studies had shed inconsistent light on the impact of insulin on the human myocard in diabetes. These studies were variable in terms of the measured effect of insulin and the time interval between insulin administration and the outcomes/parameters observed [11, 12, 16, 26]. Furthermore, studies of insulin therapy in cardiac patients had covered various groups as to their glycaemic status (healthy, type 2 diabetes, coronary artery disease), age (varying between 40.6 ± 1.8 and 65 ± 7 years), diabetes duration (nondiabetic to 11.5 ± 7.2 years), and BMI (25.2 ± 0.9 to 27 ± 2.4) [11–15].

Clinically, diastolic dysfunction is pronounced during atrial dysrhythmias, leading to pulmonary congestion. We measured the acute effect of insulin administration on cardiac function among patients with diabetes, intending to probe its potential effect on diastolic performance. Our study plots the correlation between two different blood insulin levels and their corresponding parameters of cardiac function. By maintaining euglycemia with the insulin clamp technique, skillfully performed in our laboratory, our study excluded confounding effects of hyperglycemia; thus, cardiac effects are exclusively attributed to insulin.

Baseline blood levels of insulin were within the normal range for healthy subjects. Step 1 of the EHC increased insulin to a level (145 ± 36 *μ*U/ml) which is closely comparable to the postprandial insulin levels observed in healthy persons.

At Step 1 of the EHC, both SBP and DBP blood pressure readings were significantly decreased by 5% and 4%, respectively. Concurrent to the changes in blood pressure, Step 1 of the EHC generated a significant (12%) increase in LVFS, as expected [13]. Step 2 of the EHC insulin load affected neither LVEF nor LVFS.

Diastolic LVF measurements revealed an increase in *E*′ medial amplitude at Step 1 of the EHC, indicating increased myocardial velocity during early diastole, which reflects improved diastolic LV function. Mean *A*′ increased by 20% and 27.8% during Step 1 and Step 2 of the EHC, respectively. Likewise, *A*′ lateral increased by 17% during Step 1 only of the EHC, whereas *A*′ medial increased by 30% and 44% in steps 1 and 2 of the EHC, respectively. An increase in *A*′ reflects improved left atrial contraction [27].

The A wave velocity did not change under either insulin load. However, this does not contradict the increase in *A*′, probably due to normal diastolic function at baseline. Hence, a change in the *A* wave might be practical in abnormal diastolic function cases.

Humans at rest loose an estimated 0.56 ml/min as insensible water loss (IWL) [28]. The net fluid balance in our group was 1.4 ml/min and 6.06 ml/min throughout Step 1 and Step 2 of the EHC, respectively. Rates of IWL brought the global fluid balance down to 0.85 and 5.49 ml/min during Step 1 and Step 2 of the EHC, respectively. Therefore, the net fluid balance in our group had no plausible effect on diastolic function since it fell below 18.5 ml/min, quoted as the cut-off level where volume load affects *A*′ lateral [29].

We consider the 20% increase in *A*′ during Step 1 of the EHC to be solely insulin-induced. However, in Step 2 of the EHC, a positive fluid balance was still below 18.5 ml/min [29], where its contribution to the 6% additional increase in *A*′ was not absolutely negligible.

*E* wave as well *E*/*E*′ ratio did not change under both insulin load steps, which corroborates a negligible effect of acute insulin administration upon early left ventricular diastolic filling pressure.

*E*′ medial increased by 7.5% under Step 1 of the EHC, reflecting an increase in LV compliance. The absence of an additional increase in *E*′ during Step 2 of the EHC might indicate that, under the clinically relevant blood insulin levels achieved during Step 1 (145 ± 36 *μ*U/ml), the myocard utilised its response to insulin to the fullest extent, as is the case in *A*′.

Insulin infusion of 1 mIU/kg.min brought blood insulin level to 145 ± 36 *μ*U/ml in our group, higher than its corresponding level among healthy persons (65 ± 11 *μ*U/ml). This gap was also observed in our lab, attributed to decreased insulin clearance inherent in type 2 diabetes [30, 31] and to acquired insulin resistance in hyperglycemia among type 1 diabetes subjects [32, 33].

Mechanisms that relate hyperglycemia to cardiac autonomic neuropathy (CAN) are multifactorial [34]. CAN measures were beyond our focus of interest, since we consider our study design a pivotal explorer of potential effects of acute insulin administration.

It is plausible that our subject with type 1 diabetes had no CAN as clinically judged.

A direct insulin effect on coronary blood supply could not be ruled out as displayed previously on the venous endothelium [35]; however, a metabolic study of the myocard was not a part of our protocol [36].

The role of insulin in diabetes mellitus has transformed dramatically from the glucocentric approach [37, 38] to an outcome-measures-focused approach [39–41]. Recently, insulin treatment has become well defined as the default therapy in the following cases: (1) chronic treatment of type 1 diabetes mellitus, (2) events of acute dysregulated glycaemia (hyperosmolar hyperglycemia, diabetic ketoacidosis), (3) when fresh MI is the leading clinical determinant, in diabetes and nondiabetes cases, and (4) whenever insulin is a sole default agent since other antidiabetic medications are not feasible or rather contraindicated.

Finally, studies had proven that a dose of 1 mIU/kg·min [17] or lower [14] corresponds to an effective therapeutic dose of insulin.

## 5. Limitations

The limitations of our study include the small number of patients; the nonhomogeneous group concerning the type of diabetes, its duration, age, BMI of patients, and the medications used; the normal baseline diastolic function; and the fact that humidity in the study room as well as urination volumes were not measured.

From a technical standpoint, we recommend (a) following the protocol employed here, (b) attentively tracking body weight and rates and volumes of infusates and venous sampling, (c) tracking urination volume and timing, (d) measuring room temperature and humidity scores only if feasible.

## 6. Conclusion

Acute insulin administration in patients with diabetes mellitus during concomitant normal blood glucose levels had a positive effect on some diastolic LV parameters in addition to the known improvement of systolic LV performance.

The diastolic findings shed new light on the acute effects of insulin, where therapeutic levels of blood insulin, which occur over a 2 h time course, provoke myocardial adaptive mechanisms. Insulin demonstrates its major effect upon contractile atrial function rather than LV compliance; however, some LV diastolic function parameters did show a positive response, available and attributable to therapeutic blood levels of insulin.

Our study prompts rethinking of the benefits of acute insulin administration outside routinely scheduled treatments to include cases of acute pulmonary congestion in patients with diabetes. These results may facilitate the development of insulin-based acute treatment in diabetic patients with advanced myocardial morbidity.

Larger studies are needed to establish the intriguing findings of this pilot study and to further explore the immediate, short-term, and intermediate effects of different insulin blood levels in euglycemia, in subgroups of sorted out patients with diabetes mellitus and its complications.

## Figures and Tables

**Table 1:** Patient characteristics.

Pt #	1	2	3	4	5	6	Mean ± SEM
Age (yr)	60	50	31	42	43	63	48.1 ± 4.9

Diabetes type	2	2	1	2	2	2	

Diabetes duration (yr)	20	8	9	2.5	2	45	14.4 ± 6.7

BMI	23.3	32.0	22.9	29.1	32.2	31.7	28.5 ± 1.8

Weight (kg)	73	106	75	87	91	96	88 ± 5.1

HbA1c (%)	6.3	6.2	6.0	7.6	7.1	7.0	6.6 ± 0.3

Diabetes complications	None	None	Neuropathy	Neuropathy	Neuropathy, erectile dysfunction	Neuropathy	

Diabetes therapy^*∗*^	Metformin, glibenclamide, insulin	Metformin, repaglinide. Thiazolidinedione	Insulin	Metformin, repaglinide.	Metformin	Insulin, metformin	

Other diagnoses^*∗*^	Hypertension, benign hypertrophy of prostate	Hypertension, hyperlipidemia	Hypothyroidism	Hypertension, hyperlipidemia	Peripheral artery disease, pelvic kidney	Hypertension hyperlipidemia	

Cardiac disease	None	None	None	None	None	3PTCA + 2 stents	

Tobacco use	Recent	Previous	Previous	Previous	None	None	

Concomitant medications	Ramipril	Ramipril, bezafibrate, atorvastatin	Levothyroxine	Ramipril, simvastatin, aspirin	Aspirin	Aspirin atorvastatin candesartan amlodipine beta blocker folate cyanocobalamin pyridoxine	

**Table 2:** Hemodynamic and metabolic parameters during EHC.

	Parameter	Baseline	Step 1	Step 2
Study group	Heart rate (beats/min)	68.8 ± 1.7	68.4 ± 3.2	68.3 ± 2.3
	SBP (mmHg)	138 ± 7.6	131 ± 9^*∗*^	126 ± 6
	DBP (mmHg)	80 ± 5.6	76 ± 5^*∗∗*^	77 ± 6
	Plasma insulin (*μ*U/ml)	11.6 ± 2.9	145 ± 36	1863 ± 433
	Glucose infusion rates (mg/min·m^2^)	00	79 ± 25	165 ± 38

Healthy	Plasma insulin (*μ*U/ml)	ND	51 ± 5	898 ± 131
	Glucose infusion rates (mg/min·m^2^)	ND	227 ± 24	478 ± 17

^*∗*^ *p* < 0.05 vs. baseline; ND: not determined, ^*∗∗*^*p*=0.05 vs. baseline.

**Table 3:** Cardiac echocardiographic parameters during EHC.

LVF parameter		Baseline	Step 1	Step 2
Systolic	LV EF (%)	67.6 ± 2.0	73.9 ± 3.5^#^	74.5 ± 3.8
	LVFS (%)	36.9 ± 1.6	41.3 ± 2.1^#^	43.0 ± 2.2^#,*∗*^

Diastolic	*E* wave (cm/sec)	82.3 ± 8.6	80.2 ± 8.7^§^	84.2 ± 10.0^§^
	*A* wave (cm/sec)	65.7 ± 5.2	60 ± 5.2^§^	65.4 ± 4.1^§^
	*E*/*A*	1.31 ± 0.19	1.35 ± 0.18^§^	1.3 ± 0.16^§^
	*E* deceleration time (msec)	204 ± 13.5	252.2 ± 34.9^§^	295 ± 61.8^§^
	*E*′ (cm)	10.6 ± 0.9	11.1 ± 0.9^§^	11.1 ± 1.1^§^
	*E*′ medial (cm)	8.8 ± 0.9	9.5 ± 0.8^∧^	9.0 ± 1.0^§^
	*E*′ lateral (cm)	12.3 ± 1.0	13.0 ± 0.9^§^	13.4 ± 1.2^§^
	*A*′ (cm)	7.9 ± 0.3	9.5 ± 0.5^⊗^	10.1 ± 0.4^*##,∗*^
	*A*′ medial (cm)	6.6 ± 0.3	8.6 ± 0.3^@^	9.5 ± 0.5^#^
	*A*′ lateral (cm)	9.2 ± 0.5	10.8 ± 0.6^##^	10.75 ± 0.3^#^
	*E*/*E*′	7.8 ± 0.5	7.5 ± 0.4^§^	7.6 ± 0.6^§^

Versus baseline: ^⊗^*p* < 0.01; ^∧^*p* < 0.03; ^#^*p* < 0.05; ^##^*p* < 0.005; ^@^*p* < 0.0005. Versus Step 1: ^*∗*^*p* < 0.05. ^§^*p*=non significant.

**Table 4:** Infusates volumes.

Volume	Baseline	Phase 1	Phase 2
Dextrose: water 50% (ml/120 min)	00	97.80 ± 30.6	246.0 ± 25.8
Saline 0.45% infusate (ml/120 min)^∧^	90	97.80 ± 30.6	492 ± 51.6
Insulin infusate (ml/120 min)	00	3.5 ± 0.64	19.1 ± 4.7
Blood samples for assays (ml/step)	30	30	30
Fluid balance (except IWL) (ml)	+60	+169.1	+727.1
Rest IWL^*∗*^ rates (ml/120 min)	67.2	67.2	67.2
Global net fluid balance (ml/120 min)	Deficit 7.2	101.9	659.9
Net fluid balance rate (ml/min)	—	0.85	5.49

^*∗*^IWL: insensible water loss [22].

## Data Availability

This study data can be accessed via author Ronen Bar-Yoseph; no restrictions are put on accessing the data.
